# Unleashing the synergistic effect of promising fungicides: a breakthrough solution for combating powdery mildew in pea plants

**DOI:** 10.3389/fmicb.2024.1448033

**Published:** 2024-10-30

**Authors:** Maria, Ishrat Naz, Rizwan Khan, Syed Sartaj Alam, Owais Iqbal, Shazia Akram, Nasir Ahmed Rajput, Muhammad Usama Younas, Muhammad Qasim, Ijaz Ali, Heba H. Elsalahy, Rashid Iqbal, Reem M. Aljowaie, Shahzad Munir

**Affiliations:** ^1^Department of Plant Pathology, Faculty of Crop Protection Sciences, The University of Agriculture, Peshawar, Pakistan; ^2^Department of Agriculture Extension, Government of Khyber Pakhtunkhwa, Peshawar, Pakistan; ^3^State Key Laboratory for Conservation and Utilization of Bio-Resources in Yunnan, Yunan Agricultural University, Kunming, China; ^4^Faculty of Bioscience and Technology for Food, Agriculture, and Environment, University of Teramo, Teramo, Italy; ^5^Department of Plant Pathology, University of Agriculture, Faisalabad, Pakistan; ^6^Key Laboratory of Plant Functional Genomics of the Ministry of Education/Jiangsu Key Laboratory of Crop Genomics and Molecular Breeding, Agricultural College of Yangzhou University, Yangzhou, China; ^7^Microelement Research Center, College of Resources and Environment, Huazhong Agricultural University, Wuhan, Hubei, China; ^8^Centre for Applied Mathematics and Bioinformatics, Gulf University for Science and Technology, Hawally, Kuwait; ^9^Leibniz Centre for Agricultural Landscape Research (ZALF), Müncheberg, Germany; ^10^Department of Agronomy, Faculty of Agriculture and Environment, The Islamia University of Bahawalpur, Bahawalpur, Pakistan; ^11^Department of Life Sciences, Western Caspian University, Baku, Azerbaijan; ^12^Department of Botany and Microbiology, College of Science, King Saud University, Riyadh, Saudi Arabia

**Keywords:** pea, *Erysiphe pisi*, Fungicides mixtures, synergism, disease control

## Abstract

Pea powdery mildew, caused by *Erysiphe pisi*, is a major limitation to global pea production. The emergence of fungicide-resistant pathogen populations due to frequent and injudicious pesticide application highlights the importance of exploring the synergistic properties of fungicide combinations. This study investigated the efficacy of difenoconazole, thiophanate-methyl, and sulfur, both individually and in mixtures, against powdery mildew and assessed the interaction types between these fungicides. The results demonstrated that the combination of difenoconazole, thiophanate-methyl, and sulfur was the most effective in reducing, reducing disease severity to 6.10% and minimizing conidial production on foliage. Additionally, this fungicide combination reduced conidial germination by 89.26% *in vitro* and by 87.50% in a detached leaf assay compared to the control. The treatment also positively impacted leaf chlorophyll content (55.18), green pod yield (22.21 tons ha^−1^), seed yield (12.29 tons ha^−1^), and other yield-related parameters. Although statistically significant, this ternary fungicide combination was closely followed by the binary combination of thiophanate-methyl and sulfur, which was the only combination exhibiting synergism in both laboratory and field trials with a synergy factor (SF) > 1. In conclusion, this approach offers improved disease control as part of integrated disease management (IDM) while minimizing the risk of resistant pathogen strains.

## 1 Introduction

Pea (*Pisum sativum* L.), a member of the family Leguminosae or Fabaceae (Liu et al., 2016; Rungruangmaitree and Jiraungkoorskul, 2017; Zorin et al., 2022), is a cool-season annual legume mostly cultivated for its edible seeds (Pavek, 2012). Asia is the largest producer of pea, accounting for approximately 88.8% of global pea production. Major pea-producing countries include China, India, France, the United States, Algeria, and Pakistan (FAOSTAT, 2019). In Pakistan, peas rank as the fourth most important legume, cultivated on an area of 380,000 hectares with a total production of 758,000 tons (MNFS&R, 2023).

Despite a significant increase in cultivation area and production, the per-hectare yield has declined at an annual rate of 3.6%. Several factors are responsible for this low yield, including the lack of high-yielding cultivars, the use of poor-quality, expensive seeds, and reliance on manual cultivation and harvesting practices (Aleem et al., 2020). Additionally, its production is constrained by several biotic and abiotic factors globally, including insects, nematodes, bacteria, fungi, and viruses (Ullah et al., 2020).

Among them, powdery mildew caused by *Erysiphe pisi* D. C. (Patel et al., 2017) is particularly devastating, leading to global yield losses of approximately 25–50% (Sun et al., 2016; Ikram et al., 2020). Other pathogens, such as *E. trifolii* and *E. baeumleri,* have also been associated with the disease in some regions of the world (Attanayake et al., 2010).

Most recent strategies for the control of powdery mildew include early planting, the use of resistant cultivars, and the use of fungicides (Fondevilla and Rubiales, 2012). Among all strategies, utilization of resistant cultivars is the most economical and effective measure in the control of disease (Mohapatra et al., 2016).

However, only three genes (*er*1, *er*2, and *er*3) have been identified in *Pisum* germplasm, with *er*1 being the most extensively utilized in breeding programs (Parihar et al., 2022).

The expansion of pea cultivation areas of pea varieties with the same resistance gene could facilitate the emergence of new pathogen races, potentially leading to resistance breakdown (Fondevilla and Rubiales, 2012). Subsequently, fungicides are the only panacea for the control of airborne polycyclic diseases like powdery mildew (Garcia-Figuera et al., 2024).

Fungicide control is often satisfactory, but repeated applications can be expensive, and continuous use of the same active ingredient can often lead to the emergence of resistance to fungicides (Liu et al., 2016; Iqbal et al., 2023).

Therefore, fungicides are often used in combinations to broaden their spectrum of activity, manage multiple diseases simultaneously, and exploit additive and synergistic interactions. This enhances overall effectiveness and reduces the amount of each fungicide needed without compromising performance (Dzhavakhiya et al., 2012; Akhtar et al., 2024). When fungicides are used in combination, they control the disease more effectively than when used alone, reducing the risk of resistant pathogen populations emerging (Poole and Arnaudin, 2014).

Synergy occurs when one chemical enhances the effect of another, producing a combined effect greater than the sum of their individual effects. Such chemicals exhibit synergism (Knowles, 2005) or a synergistic effect. Conversely, when the observed effect of a mixture is less than expected, it is termed an antagonistic effect (Schindler, 2017). While some researchers argue that synergism is rare in chemical mixtures (Kudsk et al., 2005; Gennings, 2010; Rodney et al., 2013), this study aims to address this research gap by evaluating: (a) the combined effects of promising fungicides on the conidial germination of *E. pisi,* pea disease, and yield and (b) the interaction types in selected fungicide combinations.

## 2 Materials and methods

### 2.1 The combined effect of fungicides on conidial germination of *Erysiphe pisi in vitro*

To investigate the effect of fungicides (difenoconazole, thiophanate-methyl, and sulfur) and their both combination such as; two-way and three-way mixture on conidial germination of *Erysiphe pisi in vitro*, a hanging drop method was employed as described earlier by Rakhonde et al. (2011). Fungicide suspensions were prepared in sterile distilled water at their recommended rates. Suspensions for the fungicide mixture were prepared for binary and ternary combinations in the ratios of 1:1 and 1:1:1, respectively. Conidia were regarded as germinated when the length of the germ tube was equal to or longer than the conidial width (Suthaparan et al., 2012). A total of 100 conidia per replicate were assessed for germination in each treatment, and then percent conidial germination was calculated. Percent conidial inhibition was calculated for each treatment using the following formula (Singh et al., 2021).


$$C=\left(X-Y\right)/X\times 100\text{,}$$

Where C is the percent conidial inhibition, X is the mean parameter of interest in non-treated control plots, and Y is the mean parameter of treated plots.

### 2.2 Combined effect of fungicides on conidial germination of *Erysiphe pisi in vivo*

For histological studies, a detached leaf assay was used under precisely controlled conditions using a modified method of Barilli et al. (2019). Individual fungicide suspensions and their mixtures were prepared by following the abovementioned procedure. Treatment application, inoculation, and percent conidial germination were performed as documented by Barilli et al. (2019). Percent conidial germination was then converted to percent inhibition.

### 2.3 Field trial

The crop was planted at the horticulture research farm at the University of Agriculture, Peshawar, Pakistan, during the cropping season of 2021–2022. The farm is situated at 34.01°N, 71.35°E, at an altitude of 350 m above sea level in Peshawar Valley. The experiment was conducted in a Randomized Complete Block Design (RCBD) with four blocks (replications) in order to counter non-homogenous conditions in the field.

Separate and independent randomization of treatments (fungicides and their mixtures, both two-way and three-way) was conducted using the Random Number Table of Fisher and Yates (1963). Seeds were sown in a two-row plot, with 12 plants per row (ridge method), in October 2021. Recommended fertilizer applications and cultural practices were followed throughout the crop-growing season.

At the eight-node stage, the experimental plants were inoculated using the method described by Parthasarathy et al. (2017) and Lim (1973) to promote natural disease development. During the second inoculation phase, the infected crop residues were spread across the field (Iqbal et al., 2017). Fungicides were applied individually as foliar sprays at the recommended doses after the onset of disease symptoms.

A two-way mixture (difenoconazole + thiophanate-methyl, difenoconazole + sulfur, and thiophanate-methyl + sulfur) and a three-way mixture (difenoconazole + thiophanate-methyl + sulfur) were prepared in ratios of 1:1 and 1:1:1, respectively. Fungicide suspensions were applied twice at two-week intervals.

Disease severity was recorded using the grid method (Feng et al., 2017) every week after the first fungicide, based on a 0–4 category scale in accordance with Javid et al. (2015). Each treatment randomly selected eight leaves per replication to assess the severity. The severity recorded for each replication was then converted to the percent severity index (% DS) according to Ji et al. (2019):


$$DS\;\left(\%\right)=\frac{\sum \left(\mathrm{No}.\mathrm{of}\;\mathrm{diseased}\;\mathrm{leaves}\times \mathrm{disease}\;\mathrm{severity}\;\mathrm{index}\right)}{\left(4\times \mathrm{total}\;\mathrm{No}.\mathrm{of}\;\mathrm{leaves}\;\mathrm{rated}\right)}\times 100$$

Percent disease control (PDC) for the experimentally conducted field trial was calculated by the following formula provided by Kamble et al. (2019):


$$PDC=\frac{PDI\;\mathrm{in}\;\mathrm{control}\;\mathrm{plots}-PDI\;\mathrm{in}\;\mathrm{treated}\;\mathrm{plots}}{PDI\;\mathrm{in}\;\mathrm{control}\;\mathrm{plots}}\times 100$$

Colby’s equation (Colby, 1967) was employed to analyze the interaction in fungicide mixtures. For binary fungicide mixtures, the following formula was used for computing the expected efficacy of the fungicide mixture (Ferry et al., 2005):


$$E_{\mathrm{Colby}}=X+Y-\frac{XY}{100}\text{,}$$

where X and Y are the percentages of disease control given by single fungicides.

For the ternary fungicide mixture, the following formula was employed for computing the expected efficacy of the fungicide mixture (Ferry et al., 2005):


$$E_{\mathrm{Colby}}=X+Y+Z-\frac{\left(XY\right)+\left(XZ\right)+\left(YZ\right)}{100}+\frac{\left(XYZ\right)}{100^{3-1}}\text{,}$$

where X, Y, and Z are the percentages of disease control given by single fungicides.

For assessing synergism, synergy factor (SF), the ratio between the observed experimental efficacy of the mixture and the expected efficacy of the mixture was calculated as follows:


$$SF=\frac{E_{\mathrm{measured}}}{E_{\mathrm{Colby}}}$$

If SF > 1, synergism is observed, whereas if SF < 1, antagonism is observed (Soller and Wedemeier, 2012).

The area under the disease progress curve (AUDPC) for each treatment was calculated based on weekly observations on disease severity (Wolf and Verreet, 2002):


$$\mathrm{AUDPC}=\overset{n}{\underset{i=1}{\sum }}[\left(Y_{i+1}+Y_{i}\right)/2\left(t_{i+1}-t_{i}\right)\text{,}$$

where Yi is the disease severity at the ith observation, ti is the time (days) at the ith observation, and n is the total number of observations.

In order to determine conidial density, eight leaves from each replication per treatment were harvested randomly. Powdery masses on leaves were scraped using a glass rod and suspended in 10 mL of sterile distilled water to prepare a conidial suspension. A hemocytometer was used to determine the number of conidia per mL of suspension as previously described using the following formula (Poudel, 2015);


$$\mathrm{Conidia}\;ml^{-1}=\mathrm{average}\;\mathrm{spore}\;\mathrm{count}\;\mathrm{per}\;\mathrm{large}\;\mathrm{square}\times 10^{4}$$

The data were log-transformed to adjust large computations.

Leaf chlorophyll content was assessed non-destructively using a portable chlorophyll meter, TYS-A (Zhejiang Top Instrument Co., LTD., Hangzhou, China). Eight leaves per replication in each treatment were randomly selected, and the SPAD value for each leaf was averaged from three measurements taken on the same leaf. Chlorophyll content was determined four times every week throughout the course of the experiment.

Data were also recorded for yield-related components, including plant height, shoot biomass, root length, number of pods vine-1, pod biomass vine-1, number of seeds pod-1, number of seeds vine-1, seed biomass vine-1, pod yield, and seed yield.

### 2.4 Statistical analysis

Analysis of Variance (ANOVA) was performed for all disease and yield parameters using the statistical package Statistics 8.1. The Fishers protected least significant difference (LSD) procedure was applied for comparing means when ANOVA showed significant variation. The GraphPad Prism version 9 was used to plot data sets into graphs, and figures were adjusted with a vector graphics editor and design software named Adobe Illustrator.

## 3 Results

### 3.1 Effect of fungicides and their combinations on conidial germination

Highly significant effects (*p* = 0.00) were evident while evaluating the inhibitory effects of fungicides and their binary and ternary combinations on conidial germination of *Erysiphe pisi* both *in vitro* and in detached leaf assay. In an *in vitro* experiment, a ternary combination of difenoconazole, thiophanate-methyl, and sulfur (DF + TM + S) strongly inhibited conidial by 7.00%, showing 89.26% inhibition compared to the control, closely followed by the binary combination of thiophanate-methyl and sulfur (TM + S) (9.80%) with a percent inhibition of 84.97 as compared to the control (Table 1). In the detached leaf assay, the least conidial germination was recorded on leaves treated with a three-way mixture of DF + TM + S (7.20%), which showed a percent inhibition of 87.50 as compared to the control, followed by a two-way mixture of TM + S (9.40%) with inhibition of 83.68% as compared to the control (Table 1). As per the Colby equation, only binary fungicide combinations of TM + S showed a synergy factor (SF) value of 1.02, indicating mild synergism in the mixture during *in vitro* and in detached leaf assays (Table 1).

**Table 1:** Effect of fungicides and their binary and ternary combinations on conidial germination of *Erysiphe pisi in vitro* and on detached leaf assay.

Treatments	*In vitro* conidial germination (48 h)				Conidial germination on detached leaf assay (24 h)			
	Conidial germination %	Percent inhibition	Expected efficacy %	SF	Conidial germination %	Percent inhibition	Expected efficacy %	SF
Control	65.20 a	–	–	–	57.60 a	–	–	–
Difenoconazole	17.60 d	73.01	–	–	17.00 d	70.49	–	–
Thiophanate-methyl	22.80 c	65.03	–	–	22.40 c	61.11	–	–
Sulfur	31.20 b	52.15	–	–	27.00 b	53.12	–	–
Difenoconazole + thiophanate-methyl	14.60 e	77.61	90.56	0.86	14.60 e	74.65	88.52	0.84
Difenoconazole+ sulfur	23.20 c	64.42	87.09	0.74	20.80 c	63.89	86.17	0.74
Thiophanate-methyl+ sulfur	9.80 f	84.97	83.27	1.02	9.40 f	83.68	81.77	1.02
Difenoconazole+ thiophanate-methyl+sulfur	7.00 g	89.26	95.48	0.93	7.20 g	87.50	94.62	0.92

### 3.2 Effect of fungicides and their combinations on disease severity and inoculum load (conidia ml^−1^)

**Figure 1:**
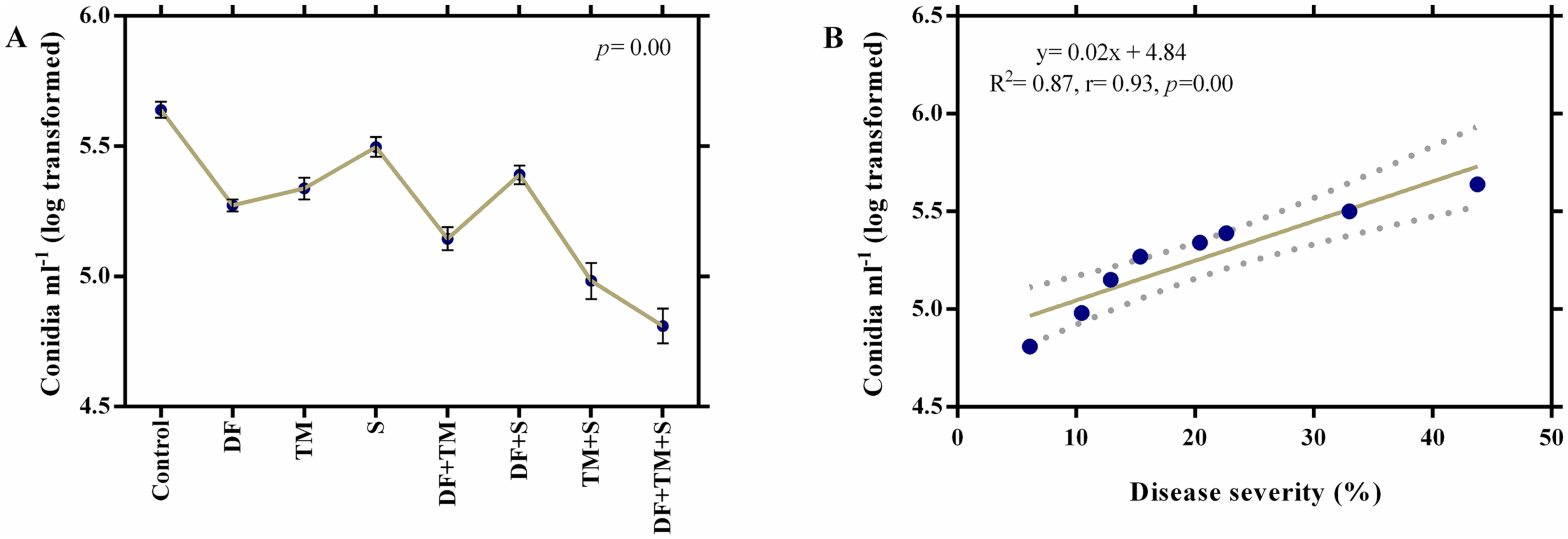
Effect of fungicides on the conidial^−1^ in the treatment. **(A)** Effect of fungicides and their binary and ternary combinations on inoculum load (Log transformed conidia mL^−1^) of powdery mildew of pea following 7 days of fungicide application. Dots and error bars represent means and standard error mean, respectively, at a significance level of 0.05. DF, TM, and S stand for difenoconazole, thiophanate-methyl, and sulfur, respectively, whereas DF + TM, DF + S, TM + S, and DF + TM + S stand for a binary fungicide mixture of difenoconazole with thiophanate-methyl, difenoconazole with sulfur, thiophanate-methyl with sulfur and a ternary mixture of difenoconazole with thiophanate-methyl and sulfur, respectively. **(B)** A regression and correlation curve between the severity of powdery mildew and conidia mL^−1^ of *Erysiphe pisi.*

Highly significant differences (*p* = 0.00) were observed while examining the effect of fungicides and their combinations (binary and ternary) on disease severity and the inoculum load (conidia ml^−1^) of pea powdery mildew under field conditions after a 7-day spray application period. The least disease severity was recorded in plots sprayed with a ternary combination of DF + TM + S (6.10%), which showed a percent control efficacy of 86.06 compared to the control, closely followed by the binary combination of TM + S (10.46%), which was 76.09% less than the control. The Colby formula showed that two-way mixtures of TM + S indicated synergism with an SF value of 1.17 (Table 2). Among all the treatments, the minimum number of conidia ml^−1^ was also recorded on plants treated with a ternary fungicide mixture (4.81), closely followed by a plot sprayed with a binary mixture of TM + S (4.98) (Figure 1A). A strong positive correlation between disease severity and inoculum load (*p* = 0.00, r = 0.93) implicated that inoculum load increases proportionately as mildew severity increases (Figure 1B). Regression analysis of disease severity and inoculum load (*y* = 0.02x + 4.84) indicated that there was a simple linear relationship between the two variables (*R*^2^ = 0.87) (Figure 1B).

**Table 2:** Effect of fungicides and their binary and ternary combinations on severity of powdery mildew of pea following 7 days of spray application.

Treatments	Disease severity (%)	Percent control	Expected efficacy (%)	SF
Control	43.75 a	–	–	
Difenoconazole	15.38 d	64.84	–	
Thiophanate-methyl	20.42 c	53.32	–	
Sulfur	33.00 b	24.57	–	
Difenoconazole+ thiophanate-methyl	12.91 de	70.49	83.59	0.84
Difenoconazole+ sulfur	22.64 c	48.25	73.48	0.66
Thiophanate-methyl+ sulfur	10.46 e	76.09	64.79	1.17
Difenoconazole+thiophanate-methyl+sulfur	6.10 f	86.06	87.62	0.98

Means in the same column followed by different letters are significantly different at *p* = 0.05.

### 3.3 Effect of fungicides and their combinations on area under disease progress curve (AUDPC)

**Figure 2:**
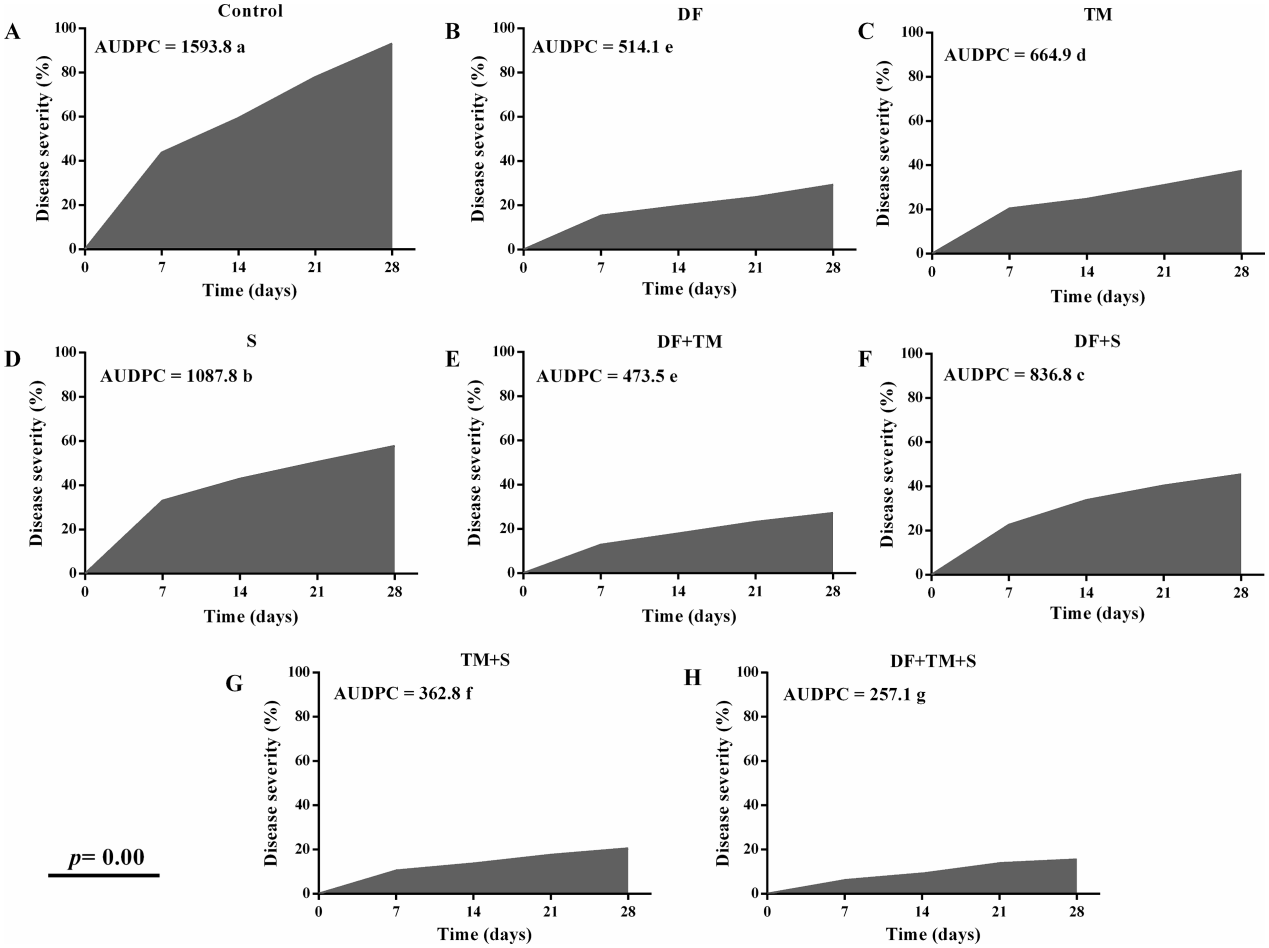
Effect of fungicides and their binary and ternary combinations on pea plants. **(A)** control with no fungicide **(B)** difenoconazole, **(C)** thiophanate-methyl, **(D)** sulfur, binary mixture of **(E)** difenoconazole and thiophanate-methyl, **(F)** difenoconazole and sulfur, **(G)** thiophanate-methyl, and sulfur, and **(H)** ternary mixture of difenoconazole, thiophanate-methyl and sulfur on Area Under Disease Progress Curve (AUDPC) of powdery mildew of pea when recorded at a weekly interval for four weeks. AUDPC means followed by different letters are significantly different at a *p*-value of 0.05.

Powdery mildew was observed across all plots from February to March 2022. Fungicide treatments significantly slowed disease progression compared to the untreated controls, as reflected in their AUDPC values (Figures 2A–H). Highly significant differences in AUDPC values (*p* = 0.00) were observed for the treatments, with disease progression monitored weekly. The plot treated with a ternary combination of DF + TM + S had an exceptionally low AUDPC value (257.1) compared to the control (1593.8) at two biweekly fungicide applications. This was closely followed by the plot treated with the binary combination of TM + S, which had an AUDPC value of 362.8.

### 3.4 Effect of fungicides and their combinations on chlorophyll content (SPAD) of pea plant

**Figure 3:**
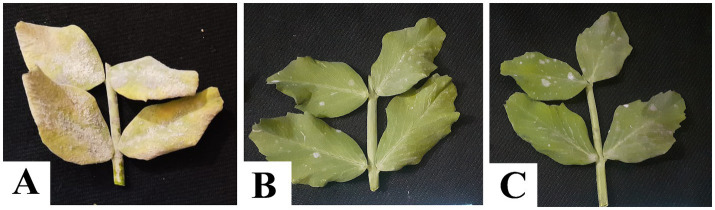
Status of chlorophyll content in plant leaves of the pea plant. **(A)** control plot with no fungicide application, **(B)** binary combination of thiophanate-methyl and sulfur, and **(C)** ternary combination of difenoconazole, thiophanate-methyl, and sulfur.

**Figure 4:**
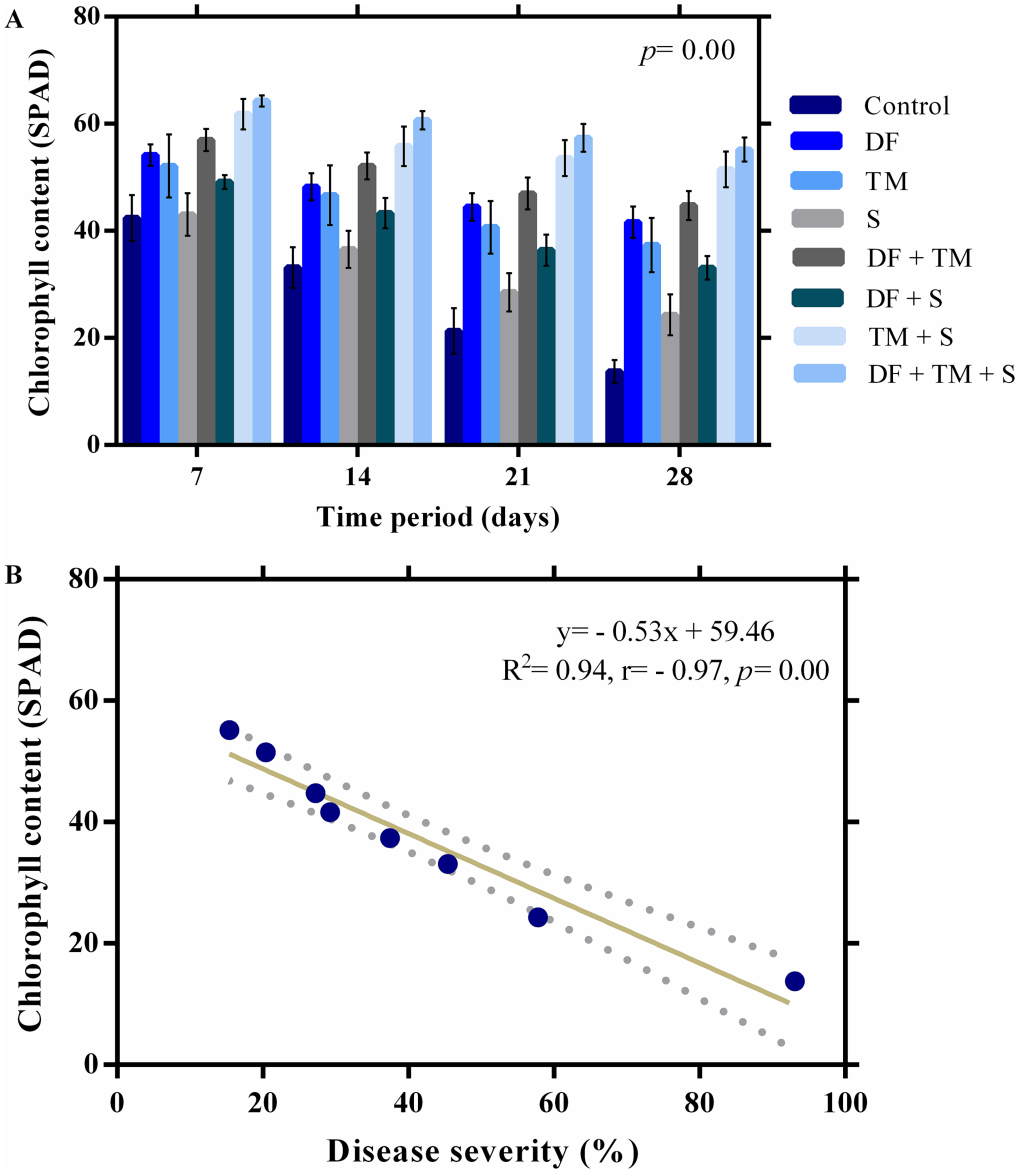
Effect of fungicides and their binary and ternary combinations on pea plants. **(A)** chlorophyll content (SPAD) of pea infected by powdery mildew. Bars and error bars show means and standard error mean, respectively, at a significance level of 0.05. DF, TM, and S stand for difenoconazole, thiophanate-methyl, and sulfur, respectively, whereas DF + TM, DF + S, TM + S, and DF + TM + S stand for binary fungicide mixtures of difenoconazole with thiophanate-methyl, difenoconazole with sulfur, thiophanate-methyl with sulfur and a ternary mixture of difenoconazole with thiophanate-methyl and sulfur, respectively. **(B)** A regression and correlation curve between powdery mildew severity and chlorophyll content following 28 days of initial fungicide application.

The leaves appeared healthy in the early stages of infection, with only slight alterations in chlorophyll content. However, it was reduced promptly with the intensification of the disease, as was evident from SPAD values (Figures 3A–C). The maximum SPAD value was observed in a ternary combination of fungicide-treated plants (64.27 SPAD), followed by a binary combination of TM + S (61.82 SPAD) after 7 days of fungicide application (Figure 4A). A strong negative correlation between disease severity and chlorophyll content (*p* = 0.00, *r* = −0.97) suggests that disease severity inversely impacted chlorophyll content in the experiment (Figure 4B). Regression analysis of disease severity and chlorophyll content (*y* = − 0.53x + 59.46) indicated a simple linear relationship between the two variables (*R*^2^ = 0.94) implicating a unit increase in disease severity value decreased chlorophyll content by 0.53 SPAD (Figure 4B).

### 3.5 Effect of fungicides and their combinations on yield and yield attributing parameters

**Figure 5:**
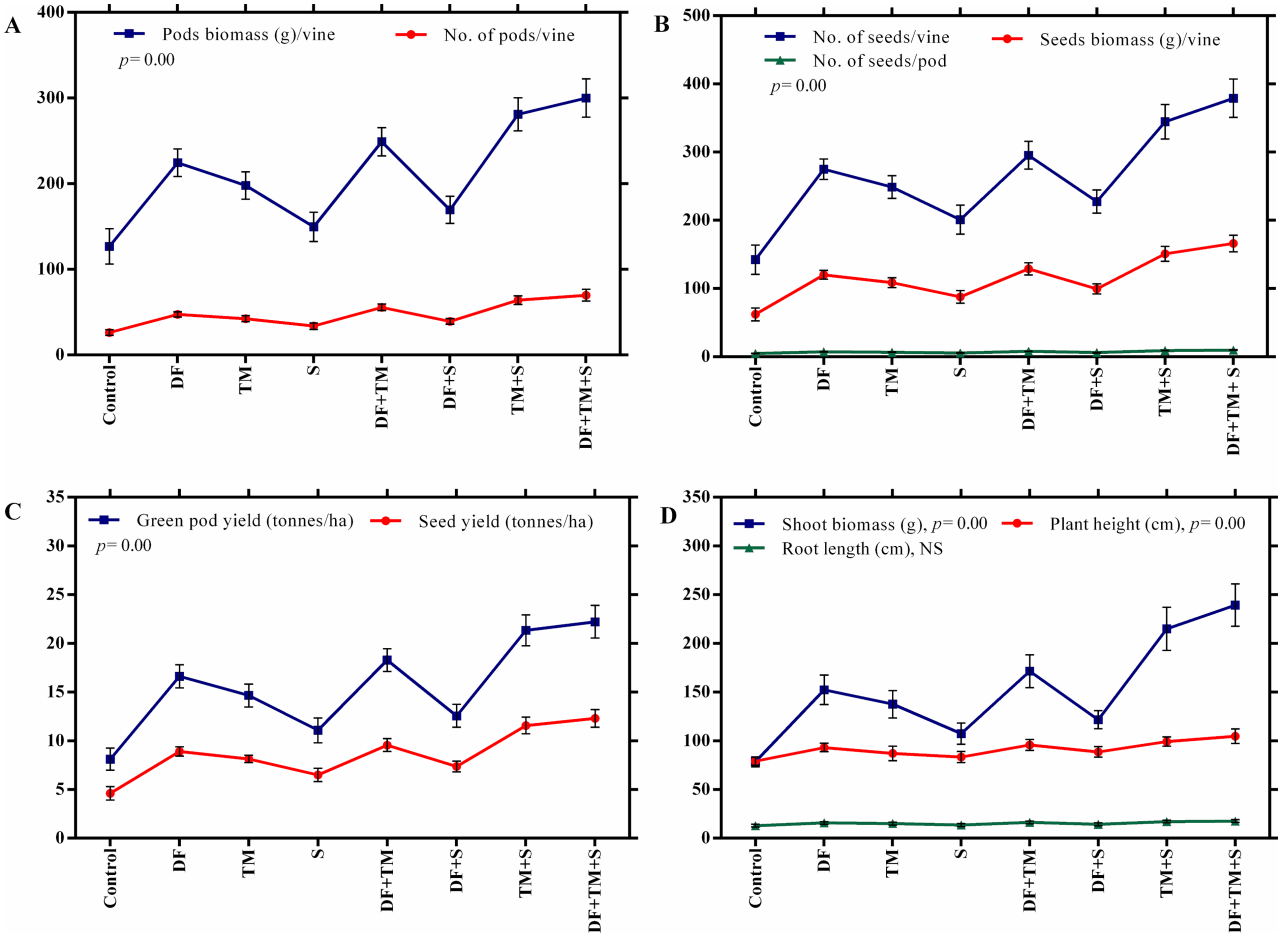
Effect of fungicides and their binary and ternary combinations on pea plants. **(A)** number and biomass of pods per vine, **(B)** number of seeds per pod, number and biomass of seeds per vine, **(C)** green pod yield and seed yield, and **(D)** shoot biomass, plant height, and root length. Symbols and error bars show means and standard error mean, respectively, at a significance level of 0.05. DF, TM, and S stand for difenoconazole, thiophanate-methyl, and sulfur, respectively, whereas DF + TM, DF + S, TM + S, and DF + TM + S stand for a binary fungicide mixture of difenoconazole with thiophanate-methyl, difenoconazole with sulfur, thiophanate-methyl with sulfur and a ternary mixture of difenoconazole with thiophanate-methyl and sulfur, respectively.

**Figure 6:**
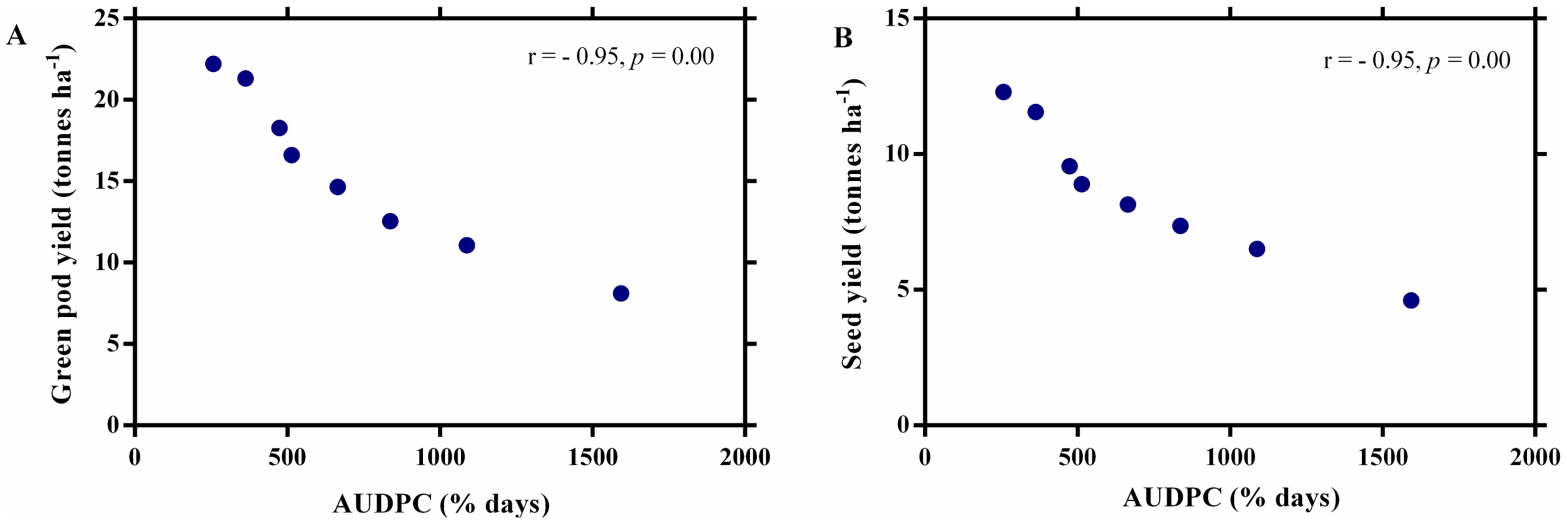
A correlation curve between the Area under the Disease Progress Curve (AUDPC) of powdery mildew with **(A)** green pod yield and **(B)** seed yield of pea.

**Figure 7:**
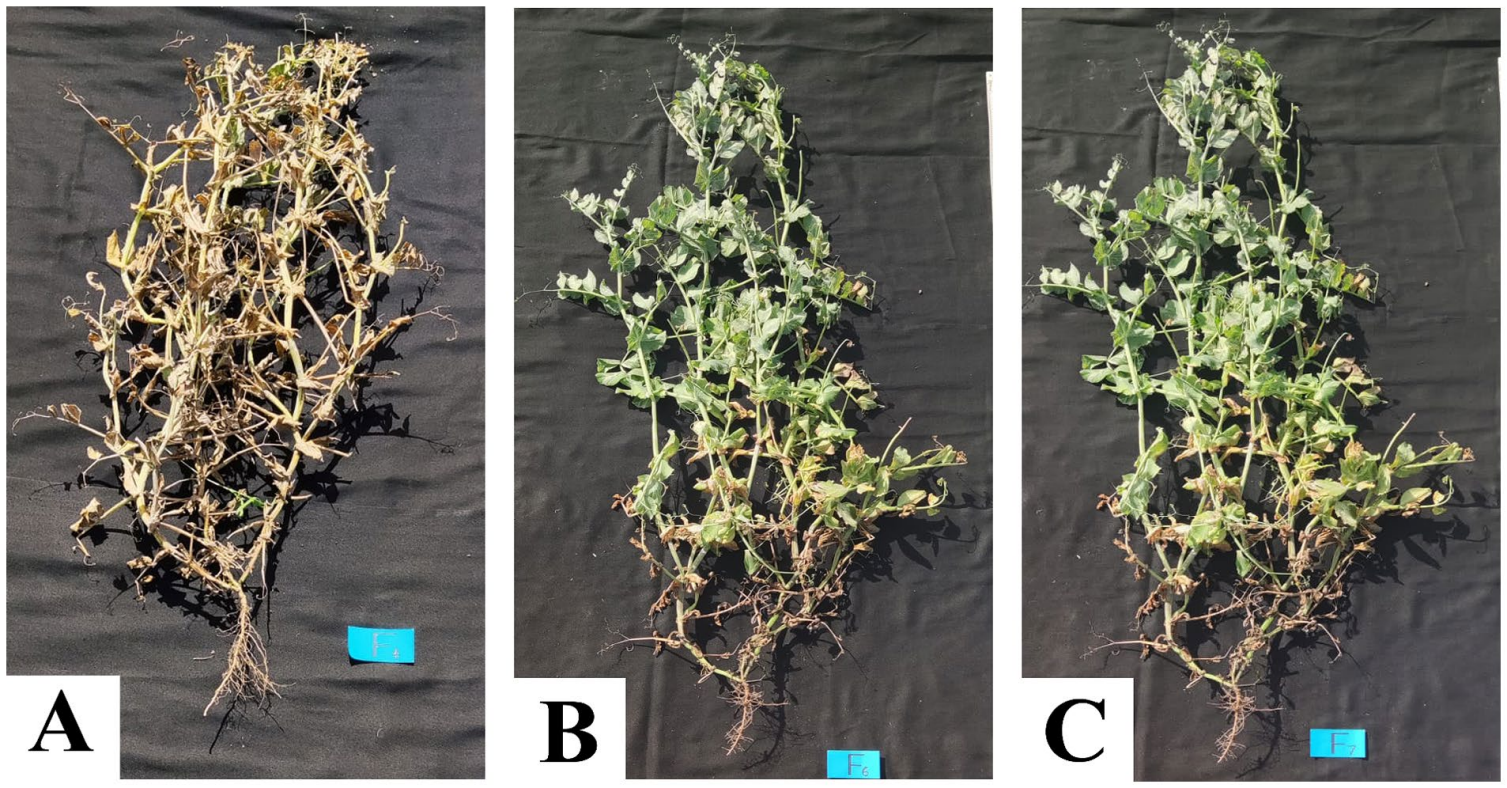
Pea plant of **(A)** control plot with no fungicide application, **(B)** treated with a binary combination of thiophanate-methyl and sulfur, and **(C)** ternary combination of difenoconazole, thiophanate-methyl, and sulfur.

**Figure 8:**
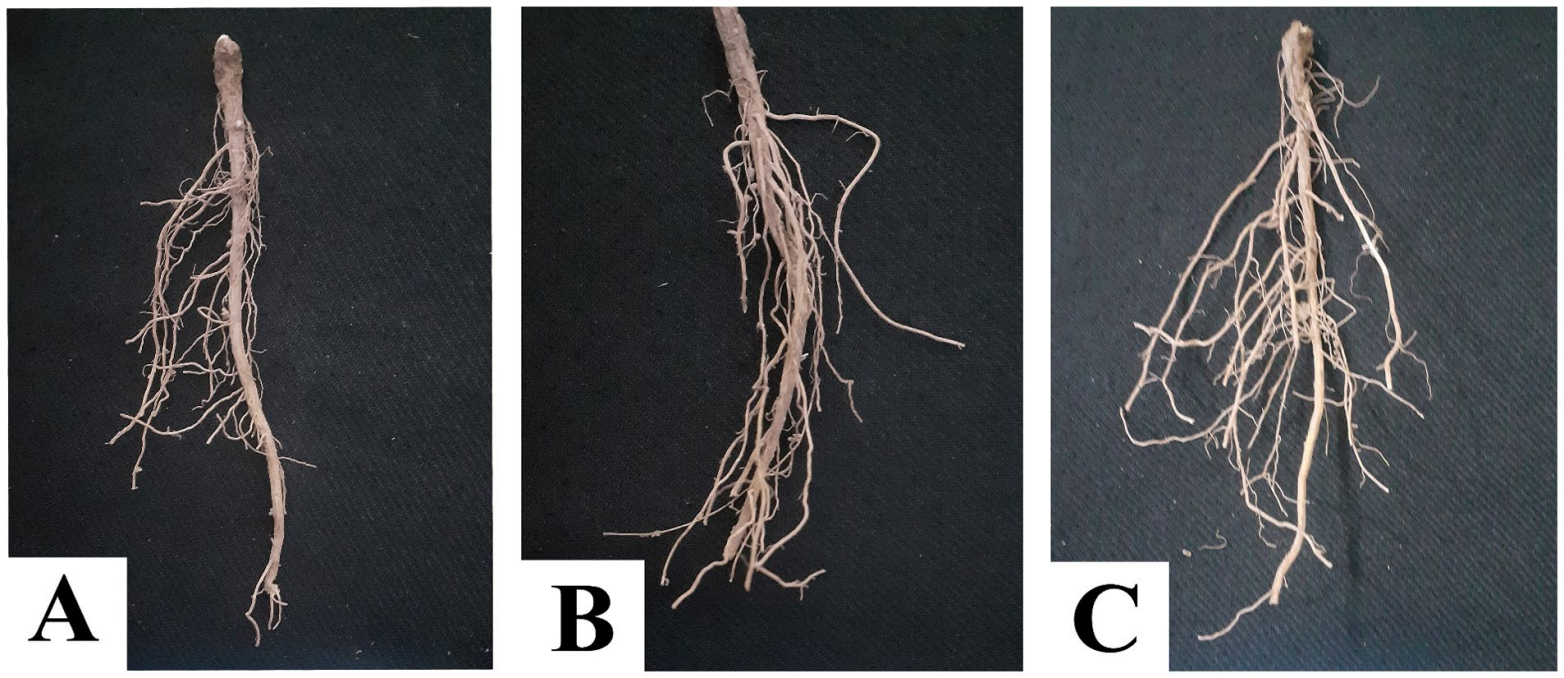
Root of pea plant of **(A)** control plot with no fungicide application, **(B)** treated with a binary combination of thiophanate-methyl and sulfur, and **(C)** ternary combination of difenoconazole, thiophante-methyl, and sulfur.

The effect of fungicides on the number of pods per vine, pod biomass per vine, number of seeds per pod, number of seeds per vine, and seed biomass per vine was observed after five pickings (Figures 5A,B). The highest values of all plant parameters were recorded in a treated plot with a ternary fungicide combination followed by a binary combination of TM + S treated plots. A similar trend was observed in green pod yield and seed yield under disease epiphytotic (Figures 5C,D). A strong negative correlation between AUDPC and green pod yield and seed yield (*p* = 0.00, *r* = −0.95) implicated that when AUDPC increases, green pod yield and seed yield decrease proportionately (Figures 6A,B). Maximum shoot biomass and plant height were observed in a treated plot with a ternary combination followed by a binary mixture of TM + S (Figures 7A–C). No significant differences were observed when evaluating the effects of the treatments on the root length of the pea plant (Figures 8A–C).

## 4 Discussion

Crop losses caused by fungal phytopathogens exceed a staggering annual total of US$ 200 billion (Dubey et al., 2021). Powdery mildew in peas, caused by *E. pisi,* is regarded as a major limiting factor for pea production globally (Barilli et al., 2019). Despite considerable efforts to develop resistant pea cultivars, synthetic chemical fungicides remain the primary method of control (Fondevilla and Rubiales, 2012).

However, repeated applications of excessive pesticide quantities have led to the accumulation of chemical residues in soil and groundwater (Itoh, 2014) and the emergence of resistant pathogen populations, thereby damaging the ecosystem and potentially posing global health risks to all living organisms (Brisbois et al., 2018). Therefore, efforts are being made to harness the benefits of general integrated disease management (Hollomon, 2015), limit pesticide doses (Van Den Bosch et al., 2011), and explore compatible pesticide mixtures that exhibit synergistic properties (Hayashi et al., 2003).

In light of this, the current experiment was designed to investigate the phenomenon of synergism in fungicide mixtures. Two systemic fungicides, with active ingredients, difenoconazole and thiophanate-methyl, and one contact fungicide, containing sulfur, were applied individually as well as in binary and ternary combinations. Since these fungicides have different modes of action, the model selected to assess their interactions is the multiplicative survival model, which is also known as Bliss independent joint action (IA) in crop science (Schindler, 2017), Independent Action, Response Multiplication, Response Addition, and Effect Addition (Cedergreen, 2014; Zhao et al., 2014). In agrochemical research, this model is associated with Colby (1967) and Limpel (1962). The Colby formula, a widely used mathematical model, is used to classify the effects of mixtures by determining the predicted impact of mixtures tested in agriculture experiments (Richer, 1987). Recently, it has been extended to multi-compound mixtures (Soller and Wedemeier, 2012).

The results of the current study revealed that synergism was observed only in a single binary combination of thiophanate-methyl and sulfur, whereas antagonistic interaction was reported in all other combinations. Synergism between chemical mixtures is rare (Cokol et al., 2011; Cedergreen, 2014). Niedobová et al. (2019) deduced that two-way mixtures of pesticides generally exhibit additive or antagonistic interaction, and synergism is an infrequent phenomenon. The mechanisms of synergy are speculative, and it might either be due to a combined effect rather than a single specific effect or due to decreased aggressiveness of the pathogen and increased concentration of components at the target site (Gisi, 1996). Onofre et al. (2021) reported that sulfur should be used in tank mixes with various synthetic fungicides, many of which are at significant risk of pathogen resistance, which might help improve its performance against powdery mildew. Devendar and Yang (2019) demonstrated that the introduction of sulfur into a biologically active molecule can dramatically modify the number of its parameters, including binding to an enzyme or target receptor, transporting the bioactive molecule from the point of application to the target site, and blocking metabolic deactivation. However, in the present study, adding a third fungicide to this combination yielded an antagonistic effect. The plausible explanation might be the incompatibility of sulfur with emulsifiable concentrates (Schilder, 2014; Sonkar and Chouhan, 2023), which is evident from the effect of their binary combination (sulfur and difenoconazole).

Similarly, it was observed in our study that difenoconazole, in combination with thiophanate-methyl, has an antagonistic effect on each other. It could be because synergistic interaction always decreases rapidly with increasing control levels of the individual components (Samoucha and Gisi, 1987) and potentially reaches low levels at high control (Gisi, 1996). The magnitude of synergy depends greatly on component toxicity, individual component ratio, concentration, and their mode of action (Levine and Borgert, 2018), as well as on the sensitivity of fungal strains to fungicides and the composition of pathogen populations (Stergiopoulos and DeWaard, 2002).

In the current investigation, the binary mixture of difenoconazole and thiophanate-methyl had a ratio of 0.84, which leans toward synergism. Levine and Borgert (2018) also reiterated that a single interaction type is implausible for delineating the precise mixture effect for all possible combinations of two agents. Thus, testing multiple ratios is beneficial for permitting a more precise estimation of mixture effects applicable to tank mixtures of pesticides used in the field. Mixtures that showed synergism reduced the dose of both active ingredients with the same control achieved by either fungicides individually (Hayashi, 2003) and can also reduce the selection pressure exerted by the resistant pathogen population (Norsworthy et al., 2012; Owen et al., 2014). Synergistic interaction is particularly beneficial when resistance exists in one mixture partner (Thind and Hollomon, 2018). Mixtures can also extend the spray interval time (Hayashi, 2003), resulting in fewer applications than fungicides that are applied individually (Levine and Borgert, 2018).

A significant reason for these findings is that the most effective modern synthetic fungicides used for powdery mildew control are prone to insensitivity development (Sombardier et al., 2009). This is particularly true for fungicides with systemic site-specific activity (Thind and Hollomon, 2018), which are popular due to their non-toxicity to the environment and non-target organisms (Beffa, 2004), and broad-spectrum fungicides, which are also used for fungal diseases other than powdery mildew on crops (Whitaker et al., 2018). In such cases, the pathogen population can easily overcome their efficacy by undergoing subtle genetic changes, resulting in a complete loss of disease control that cannot be regained by using higher rates or more frequent fungicide applications (McGrath, 2004). High fungicide application frequency required to suppress powdery mildew often results in rapid resistant phenotype selection (Daughtrey and Benson, 2005). As a result, fungicide resistance can be managed by minimizing the use of “at risk” fungicides, applying at the manufacturer’s recommended rates and application interval, or using the alternation of “at risk” fungicides with chemical groups of different modes of action (McGrath, 2004) or in combination with other fungicides (low-risk fungicides). Mixtures offer an advantage compared to alternation since there is no need to delay the application of the high-risk fungicide, and the resistant strains do not rise to high frequencies, lowering the risk of its further spread (Mikaberidze et al., 2014). Generally, fungicide interactions in mixtures result in novel phenomena that cannot be inferred from the single compounds alone (Ammermann et al., 2009; Martin et al., 2021). In all likelihood, placing a multi-site inhibitor in a mixture with a site-specific inhibitor is a better approach to lowering the risk of resistance development and broadening the antimicrobial spectrum (Hayashi, 2003; Thind and Hollomon, 2018). In the present study, a binary mixture of thiophanate-methyl (site specific) and sulfur (multisite) exhibited synergism, thus beneficial in insensitivity development and improving disease control.

## 5 Conclusion

Pea powdery mildew, caused by *Erysiphe pisi*, is a significant pathogen responsible for considerable economic losses in pea cultivation worldwide. Various disease management strategies, such as general integrated disease management, dose limitation, and alternation of “fungicides with different modes of action, are commonly employed to combat this issue. Combining “at risk” fungicides with low-risk alternatives, utilizing mixtures, and exploring new pesticidal molecules are key strategies for improving crop protection. Our research contributes to this field by exploring the synergistic effects of fungicide mixtures to minimize usage without sacrificing efficacy. This approach not only reduces the dose and application frequency but also helps mitigate the risk of resistance development in pathogens.

## Data Availability

The raw data supporting the conclusions of this article will be made available by the authors without undue reservation.
